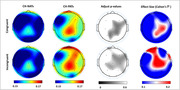# Brain Functional Connectivity During Stroop Task and CSF Amyloid/tau in Cognitively Healthy Individuals

**DOI:** 10.1002/alz.090859

**Published:** 2025-01-09

**Authors:** Abdulhakim Al‐Ezzi, Natalie Astraea, J Yu, Xiaomeng Wu, Alfred N. Fonteh, Yafa Minazad, Robert A Kloner, Xianghong Arakaki

**Affiliations:** ^1^ Huntington Medical Research Institutes (HMRI), Pasadena, CA USA; ^2^ Huntington Medical Research Institutes, Pasadena, CA USA; ^3^ University of Southern California, Los Angeles, CA USA; ^4^ Southern California Neurology Consultants, Pasadena, CA USA

## Abstract

**Background:**

At the pre‐clinical stages of Alzheimer’s disease (AD) development, the accumulation of amyloid‐β (Aβ) and tau induces neural toxicity, synaptic dysfunction, and excitation/inhibition instability of neural network activity, leading to cognitive decline. However, the effects of Aβ/tau accumulation on electroencephalography (EEG) functional connectivity (FC) in cognitively healthy (CH) individuals during a cognitive challenge have not been elucidated. Therefore, the main objective of this work is to evaluate the association between Aβ/tau level and brain FC during a cognitive challenge in CH individuals.

**Method:**

Established EEG data was recorded using a 21‐EEG sensor headset (Wearable Sensing, DSI‐24) during a cognitive challenge (Stroop interference test). Amyloid‐β and total‐tau proteins were measured from cerebrospinal fluid (CSF) using electrochemiluminescence. We assessed FC at sensor level using Partial Directed Coherence (PDC) from EEG data of CH participants including 22 normal CSF Aβ/tau ratio (CH‐NAT) and 24 pathological CSF Aβ/tau (CH‐PAT). For each trial type (congruent or incongruent) in the Stroop task and each of 19 sensors, linear regression was used to estimate the difference in mean FC between CH‐NAT and CH‐PAT, while controlling for age, gender, and years of education. The Benjamini–Hochberg procedure was used to control the false discovery rate below q = .1.

**Result:**

Compared with CH‐NATs, CH‐PATs exhibit higher causal connectivity at specific sensors which each had an estimated local effect size index Cohen’s f^2^ >0.15, and putatively represent brain regions including medial prefrontal cortex (Fz, Fp1, and F8), central cortex (Cz and C4), and posterior cingulate cortex (Pz). For each of the same sensors, the estimated difference between CH‐NAT and CH‐PAT had an adjusted P value below .1.

**Conclusion:**

These findings suggest a potential association between the CSF Aβ42/tau pathology and differences between individuals in brain connectivity during a cognitive challenge. These differences may involve compensatory mechanisms as the brain adapts to the amyloid/tau pathology. These results provide potential insights into the neurobiological implications of varying CSF amyloid/tau on brain networks. The areas with estimated medium‐to‐large effect sizes are favorable to doing a study with a lower significance level to confirm the findings.